# Long-Acting Injectable Therapy for People with HIV: Looking Ahead with Lessons from Psychiatry and Addiction Medicine

**DOI:** 10.1007/s10461-022-03817-z

**Published:** 2022-09-05

**Authors:** Gabriel G. Edwards, Ayako Miyashita-Ochoa, Enrico G. Castillo, David Goodman-Meza, Ippolytos Kalofonos, Raphael J. Landovitz, Arleen A. Leibowitz, Craig Pulsipher, Ed El Sayed, Steven Shoptaw, Chelsea L. Shover, Michelle Tabajonda, Yvonne S. Yang, Nina T. Harawa

**Affiliations:** 1 Division of General Internal Medicine & Health Services Research, David Geffen School of Medicine at University of California Los Angeles, Los Angeles, CA USA; 2 Department of Social Welfare, UCLA Luskin School of Public Affairs, Los Angeles, CA USA; 3 Center for Social Medicine and Humanities in the Department of Psychiatry and Biobehavioral Sciences, University of California Los Angeles, Los Angeles, CA USA; 4 Division of Infectious Diseases, David Geffen School of Medicine at University of California Los Angeles, Los Angeles, CA USA; 5 Department of Psychiatry and Biobehavioral Sciences, David Geffen School of Medicine at University of California Los Angeles, Los Angeles, CA USA; 6 Greater Los Angeles Veterans Healthcare Administration, Los Angeles, CA USA; 7 UCLA Center for Clinical AIDS Research & Education, David Geffen School of Medicine at University of California Los Angeles, Los Angeles, CA USA; 8 Department of Government Affairs, APLA Health, Los Angeles, CA USA; 9 Department of Pharmacology, Touro College of Medicine, New York, NY USA; 10 Department of Family Medicine, David Geffen School of Medicine at University of California Los Angeles, Los Angeles, CA USA; 11 Department of Health Policy and Management, Fielding School of Public Health, University of California Los Angeles, Los Angeles, CA USA; 12 Semel Institute for Neuroscience and Human Behavior, David Geffen School of Medicine at University of California Los Angeles, Los Angeles, CA USA; 13 Department of Psychiatry, Charles R. Drew University of Medicine and Science, Los Angeles, CA USA; 14 Division of General Internal Medicine & Health Services Research, David Geffen School of Medicine at UCLA, 1100 Glendon Ave., Suite 850, Los Angeles, CA 90024 USA

**Keywords:** HIV treatment, Long acting injectable medication, Antiretroviral medication, Addiction medicine, Psychiatry, Implementation barriers and facilitators, Cost

## Abstract

Long-acting injectable antiretroviral medications are new to HIV treatment. People with HIV may benefit from a treatment option that better aligns with their preferences, but could also face new challenges and barriers. Authors from the fields of HIV, substance use treatment, and mental health collaborated on this commentary on the issues surrounding equitable implementation and uptake of LAI ART by drawing lessons from all three fields. We employ a socio-ecological framework beginning at the policy level and moving through the community, organizational, interpersonal, and patient levels. We look at extant literature on the topic as well as draw from the direct experience of our clinician-authors.

## Introduction

Forty years into the HIV pandemic, advances in antiretroviral treatment have dramatically reduced HIV-related morbidity and mortality for people with HIV (PWH) who adhere to daily oral antiretroviral medications (ART) [1]. However, many people do not adhere to the daily regimen and thus the benefits of these advances in treatment have not been sufficient to then the epidemic nor distributed uniformly. Some key populations disproportionately do not reach or maintain the critical milestone of HIV suppression: undetectable viral levels in blood plasma. In 2019, 81.3% of PWH were linked to care within 1 month of their diagnosis, but only 65.5% of those linked to care were virally suppressed by 6 months following diagnosis [2]. This suggests that the other 34.5% of PWH were either not prescribed ART, were not adherent to the regimen, or discontinued treatment.

Long-acting injectable (LAI) formulations of medications may help fill the gap by improving uptake, adherence to, and persistence of treatments and preventives for chronic conditions like HIV. The longer-standing experiences of delivering LAIs for other chronic diseases offer insights into the delivery and use of newly available LAIs for HIV prevention and treatment. The latter is also referred to as long-acting injectable antiretroviral therapy or LAI ART for short. Lessons learned from the longer-standing use of LAIs in the fields of mental health and addiction treatment hold particular relevance given that they treat a population that includes people with HIV (PWH). Moreover, conditions from the three fields strongly overlap with one another and have been studied as a *syndemic*, with other conditions often grouped in as well [3–5]. A large cohort study of HIV-positive adults found that nearly half had a history of substance use disorders (SUDs) [6]. The prevalence of mental health disorders among PWH is several times higher than the general population [7] and, if anything, conditions like depression are *underdiagnosed* in HIV care [8]. Furthermore, the prevalence of HIV is substantially higher among adults with serious mental illnesses such as psychosis and bipolar disorder than those without mental illness [7]. Drug and alcohol dependence are associated with decreased access to and use of healthcare, reducing the likelihood of being prescribed ART (and adherence once prescribed) [9]. Similarly, untreated mental illness results in worse outcomes for treatment of both HIV infection and SUDs [9].

In January 2021, the US Food and Drug Administration (FDA) approved the first LAI ART, cabotegravir/rilpivirine. We employ a socio-ecological framework to discuss issues surrounding the implementation of LAI medications for all three types of conditions and explore ways to maximize potential benefits for HIV [10]. We start with some background on the current state of LAI ART, followed by a discussion that moves from the *policy level through the community, organizational, interpersonal, and patient levels*. This commentary’s authors include psychiatrists, addiction specialists, HIV providers, health services researchers, and health policy experts. We draw from our own professional experiences and the extant literature to discuss barriers, facilitators, and issues of costs and ethics related to LAIs in the fields of mental health, SUD, and HIV.

A note about scope: when discussing LAIs in psychiatry to address mental illness, our focus is on anti-psychotic medications and for SUDs, on the treatment of opioid use disorders (OUDs). LAI antipsychotics have the longest history, with the first generation of these medications introduced in 1966, and the first second-generation LAI antipsychotic approved starting in 2003 [11, 12]. The FDA first approved extended-release naltrexone in 2010 for relapse prevention in people with OUD following detoxification [13] and extended-release buprenorphine in late 2017 for those who had initiated treatment with transmucosal (absorbed through mucus membrane) buprenorphine [14].

## Background

### Current HIV Treatment Landscape

LAI cabotegravir/rilpivirine is currently the only FDA-approved injectable ART for patients who have reached viral suppression using oral medication. Initiating and treating a patient on the regimen currently includes the following steps: (1) assessing the patient for clinical appropriateness, (2) beginning the treatment regimen with an oral version of the injectable regimen (also called a “lead-in period”) to establish tolerability, (3) if tolerated, administering a large initial dose (loading dose), and (4) then providing ongoing smaller subsequent doses at 1–2 month intervals.

An in-depth discussion of potential complications is beyond the scope of this paper, but includes potential drug interactions, the need to switch back to oral medications should the injections be delayed from their regular schedule (called “bridging”), and the risk of viral resistance should treatment delays become too long or frequent. Some infrastructural considerations moving forward include developing the capacity for home-based or point-of-care delivery (e.g., pharmacies or other businesses) of LAI treatments [15]. Also important is the harmonization of tracking records to ease implementation across multiple access sites. Several entities, including the manufacturers of cabotegravir/rilpivirine, have ongoing efforts to increase provider awareness about injectable ART options, as well as to discuss and address potential staffing, tracking, patient counseling, and medication administration needs.

In addition to challenges to ART uptake and adherence outlined at the beginning of the introduction, documented disparities in HIV treatment outcomes include differences by age and among racial/ethnic, sexual, and gender minorities [16]. Individuals facing adverse social and structural barriers (e.g., housing instability) [17, 18], poverty [19], criminal justice involvement [20], HIV-related stigma [21], intimate partner violence [22], and SUDs [23] are at increased risk for suboptimal retention in care and medication adherence. The introduction of LAIs ART, which reduces the need for frequent dosing, increases the potential for discreet treatment, and alleviates “pill fatigue,” thus has the potential to help reduce those disparities. However, LAIs could also introduce new challenges and new barriers for these marginalized groups that must be addressed proactively. Examining lessons learned in implementing LAIs for the treatment of SUDs and mental illness provides opportunities to identify and address such challenges and barriers early in the rollout of LAI ART.

## Policy

### Cost

*LAIs for mental illness and OUD are expensive* relative to oral treatments and cost is often a structural barrier. Nevertheless, they have been found to be cost-effective. That the medications are both *costly* and *cost-effective* underlines the importance of committing up-front investment to realize the benefits of this intervention and to ensure that costs are distributed equitably.

Anti-psychotic LAI medications are associated with better health outcomes when compared to oral formulations [24]. The higher initial cost for anti-psychotic LAIs is offset by lower subsequent costs for medical care through mechanisms like lower hospitalization rates and shorter inpatient stays [25, 26]. The lower social costs of well-controlled schizophrenia include reduced involvement in the criminal legal system, substance abuse, and violence [27].

*The cost of LAI ART and several first-line oral HIV medications is comparable* [28]. According to the U.S. Department of Health and Human Services, the monthly average wholesale price of LAI cabotegravir/rilpivirine ranges from $4752 to $7218 depending on the dose, which amounts to $43,308 per year for bi-monthly injections or $57,024 per year for monthly injections (excluding oral lead-in and initiation injections). In comparison, the annual average wholesale price of bictegravir/emtricitabine/tenofovir–one of the most commonly prescribed oral HIV medication combinations—is $48,876 [29]. This formulation is still under patent, and branded versions of antiretrovirals are substantially more expensive than generic versions [30]. Thus, as patents for oral HIV medications continue to expire, the cost differential between LAIs and oral medications will grow. Still, LAIs may remain cost-effective given greater beneficial impacts on adherence and viral suppression [28]. Because youth experience particular challenges adhering to ART for both treatment and prevention [31–33], cost analyses should estimate relative cost and benefits specific to young PWH.

*There are important distinctions between the potential benefits of LAI ART as compared to LAIs for mental illness and SUD*. ART adherence has been estimated at 63.4% worldwide and 74.1% in the U.S. [34, 35]. This adherence rate is higher than estimates for patients taking oral buprenorphine (37.1–41.3%) and among patients with schizophrenia taking antipsychotics (31.5–68.7%) [24, 36]. Given the differences between the medications and the conditions they are intended to treat, the variation in adherence numbers are expected. The types of costs associated with undertreated HIV and undertreated mental illness and SUD also differ. HIV, as distinct from substance use and schizophrenia, is a communicable illness with an estimated, discounted lifetime treatment cost of $420,285 in 2019 U.S. dollars, on average [37]. Poor adherence increases the risk of onward horizontal and vertical transmission, as it increases levels of circulating virus above the level that prevents HIV transmission to sexual partners and from birthing parents to their offspring [38, 39]. For all three specialties, documented costs include hospitalization, whose cost is borne by third-party systems such as public payers. For SUDs and mental illness, additional costs include engagement with the criminal legal system, fatal overdose, and increased risk of victimization from (or perpetration of) violence [40–45]. For individuals dually diagnosed with mental illness and SUD, social costs may be compounded.

### Insurance Coverage

Medicare considers ART medications a “protected drug class” that must be included in Medicare Part D formularies. However, while some Medicare Advantage plans may opt to cover cabotegravir/rilpivirine under Part D, it will likely be covered more often under the less protected Part B as a physician-administered drug. Additionally, because ART medications are quite costly, insurers may institute cost-containment conditions, including requirements for prior authorization, patient cost sharing, step therapy, and formulary exclusions. These practices can impede appropriate LAI prescribing [46].

Cost-containment practices may vary depending on whether medications are prescribed under the medical benefit for injections administered in the medical provider’s office or under the pharmacy benefit. Utilization management is more common for limiting the use of high-cost medications under a plan’s pharmacy benefit [47]. Although the pharmacy benefit typically covers oral products and self-administered injectables, more provider-administered products are starting to fall under the pharmacy benefit [48].

Experience with insurance coverage for LAI antipsychotic medication delivered at the point of care suggests that when the LAIs are insured under the plans’ medical benefit, providers may have to pay in advance and stockpile the medications before injecting them and then seek reimbursement from third-party payers as they are used. This is considered “buy-and-bill” purchasing. This process can be complicated and cost-prohibitive due to the high up-front costs. This may present a barrier to HIV LAI prescribing, particularly for clinics that cannot afford the high upfront costs that come with cabotegravir/rilpivirine.

PWH who lack public or private insurance, many of whom are undocumented or live in Southern states that have not expanded Medicaid, can receive HIV treatment and wraparound services through the Ryan White HIV/AIDS Program. However, Ryan White covered providers are largely unavailable in rural parts of the country, putting the high-cost HIV medications out of reach for many PWH, whether oral or injectable. A report by the South Carolina Rural Research Center found that 95% of rural U.S. counties lacked a Ryan White provider [49]. A disparity in provider access between urban and rural areas may widen geographic differences in LAI access (which cannot be delivered in the mail like oral pills), leading to disparities in HIV outcomes. This dynamic warrants improvement in rural access to comprehensive HIV care.

*The potentially higher costs of LAIs for HIV may dissuade correctional institutions from providing them, and those patients who do access them during incarceration may struggle to do so on release*. The period following incarceration is characterized by a disruption of care, including for HIV [50]. PWH with SUD are at particular risk for negative outcomes during reentry, with even a brief incarceration strongly associated with virologic failure among people who use injection drugs [51]. According to a 2015 systematic review, the number of PWH receiving ART while incarcerated varies widely, but on average 65% received it during incarceration, a number that dropped to just 37% following release [52]. LAIs, if administered before release, could help cover the period immediately following re-entry into the community [53]. But policy barriers remain; health coverage during periods of incarceration in the U.S. is complicated by the Federal Medicaid Exclusion Act. Medicaid is not available to otherwise-qualified individuals while incarcerated, except for those who are hospitalized overnight while in custody, necessitating correctional institutions to finance medications themselves. U.S. carceral institutions are also not eligible for 340B pharmaceutical pricing, which substantially increases the costs that they pay for HIV medications compared with those providers that treat these same populations in the community [54]. The situation is not, however, static. Many states have moved to suspend, rather than terminate Medicaid during incarceration, facilitating reinstatement, and some local initiatives work to facilitate access to Medicaid during the transition from incarceration to reentry [55]. In 2019, 90% of state prison systems had at least one prison where buprenorphine, methadone, or naltrexone is available, and 62% have at least one prison that offers all three medications [56].

## Community

### Stigma and Marginalization of People Living with Specific Health Conditions

Although HIV, mental illness, and SUD are biologically and experientially distinct, all are chronic, highly stigmatized conditions that disproportionately affect populations that have otherwise experienced marginalization. Higher burdens or worse outcomes of these conditions are documented among Black, Latinx, and Indigenous populations, sexual and gender minority communities, people who are incarcerated, and those experiencing poverty [57]. Care of patients with these conditions has historically been fraught, as many clinicians and systems of care have contributed to this stigma, marginalization, and mistreatment [57]. Furthermore, these conditions are not mutually exclusive. It is estimated that 31% (95% CI 28–34%) PWH have moderate-to-severe levels of depression [58] and 25% (CI 95%, 21–30%) have an anxiety disorder [59]; however, the estimated prevalence of psychoses that are treatable with LAIs has only been published in studies with small, non-representative samples of PWH. The literature showing elevated frequencies of substance use in PWH is extensive [60] and, in recent years, has included outbreaks among people who inject opiates [61].

The U.S. has always had a low rate of use of LAI antipsychotics compared to European countries [62]. LAIs in the U.S. have likely been preferentially used in settings where clinicians are concerned about low rates of treatment adherence and persistence and in settings of inadequate treatment infrastructure, comorbid substance abuse, or housing instability. Differences in administration by race have also been identified. Around the year 2000, researchers found LAI antipsychotics to be prescribed to Black patients in the U.S. at higher rates than other patients, regardless of the clinical setting [63, 64]. Although a recent study in a large US county mental health system did not find this difference [65], a recent United Kingdom-based study also found increased LAI use among Black patients [66]. Potential racial differences in the use of LAI antipsychotics contribute to concerns about coercion and ethics, including involuntary treatment and the prioritization of clinical or social goals over patient autonomy [57]. The often legally mandated use of LAI antipsychotics, including for court-ordered treatment and carceral settings, makes navigating these issues more challenging for concerned providers and health systems. The frequent association of LAI antipsychotics with coercion and involuntary treatment is unfortunate given that data supports better outcomes compared with oral antipsychotics, and that LAI formulations can be used in a collaborative manner that improves patient autonomy, satisfaction, and quality of life [67]. We explore issues of coercion further below.

### Medical Mistrust

While LAI ART treatment lacks the troubled history of LAI antipsychotics, concerns related to potential coercion and malfeasance remain. Since the beginning of the AIDS pandemic, outreach, prevention, and treatment have faced significant mistrust from the communities most affected. Often rooted in historical and current experiences of racism, homophobia, and stigma [68, 69], medical mistrust and conspiracy beliefs are rational responses to engaging sometimes hostile systems of care that are embedded within larger societies infused with these ideologies [70]. Mistrust in the setting of LAI ART has been perceived as a barrier, particularly for Black patients, and community engagement is critical for effective message development and successful implementation [71]. Principal among these concerns was the idea that HIV itself and later that HIV testing, treatment, and prevention efforts (including needle exchange and condoms) were intended to rid society of unwanted members [72–78]. Conspiracy beliefs that people were infected with HIV while having blood drawn for HIV testing or that HIV was introduced into African populations through vaccination campaigns, rather than emerging naturally from chimpanzee species, hampered HIV testing campaigns early in the U.S. pandemic [79]. Recent surveys indicate that substantial percentages of Black Americans continue to harbor conspiracy beliefs, though the association of mistrust with preventive behaviors is complex [80].

## Organizational

### Infrastructure

*Infrastructure issues vary between the three specialties*. Capacity is critical to administering LAIs and includes addressing needs related to training and staffing, storage, and in some cases, refrigeration [81]. Many mental health providers lack the nursing staff to directly deliver injections. Although some pharmacies work with such providers so that they are set up to administer anti-psychotic LAIs, current partnerships of this kind do not appear to be widespread. Private group practices that do not regularly staff nurses also may pool resources to hire a nurse to administer injections on a set day of the week and to purchase equipment such as refrigerators to store medications [81]. MOUD treatment happens in opioid treatment programs, inpatient/outpatient rehabilitation centers, and primary and psychiatric care clinics in addition to emergency rooms. Most of these settings can offer injections. However, some may lack the refrigeration required to store cabotegravir/rilpivirine. In the HIV context, therefore, healthcare providers may have the capacity to administer LAIs for ART but still need to determine how to address the need for medication storage and more frequent visits among patients on LAIs compared with those on daily oral treatments. The HIV care system also benefits from existing networks of specialty care delivery, such as the Ryan White HIV/AIDS Program and large provider networks (e.g., AIDS Healthcare Foundation) that might facilitate the widescale rollout of LAIs.

The increasing role that pharmacies have played in addressing the COVID-19 pandemic underscores the importance of pharmacies in successful LAI implementation. In the United States, pharmacists already perform injections for a wide variety of medication classes, including antipsychotics and long-acting opioid agonists [82]. To both increase capacity and reduce the time and travel associated with obtaining LAI, providers should identify pharmacies and community-based drugstores that have personnel able to perform injections, and policymakers should consider some manner of financial incentive for LAI administration by pharmacists. Restructuring of clinic schedules, electronic health record systems, and reimbursement structures may facilitate the increased frequency of short visits required for LAI treatment in the HIV context.

### Coercive Environments

*The use of LAIs differs by specialty when it comes to use in carceral settings*. The use of LAI antipsychotics has been explored in some locales and jail settings, where the medication has been set as a condition of supervised release [83]. Involuntary administration of ART is rare in this and other settings. To our knowledge, there are no instances of mandated HIV treatment in carceral settings; hence, individuals who have been mandated or forced to use LAI antipsychotics may struggle to accept or understand the idea of voluntary use of LAI ART. Furthermore, given that institutionalized patients have control over a few other aspects of their daily lives, the ability to accept or refuse a daily medication may be one of the few ways to enact bodily agency. Injection with a treatment that endures in the body for weeks to months relinquishes that control [57, 84].

SUDs are common among people in jails and prisons, including those with HIV [85]. Interest is growing in the use of LAI for SUD in correctional settings, with naltrexone favored over buprenorphine by many leaders in the criminal legal system [86]. Although at least one study has shown naltrexone to support the achievement of HIV viral suppression relative to placebo among PWH following reentry [87], both the requirement for detoxification before naltrexone initiation and the treatment’s mechanism of action can increase the risk of fatal overdose [88]. Given the multiple inequities contributing to the overrepresentation of Black people in correctional facilities, the preference for naltrexone in this setting likely contributes to large disparities in buprenorphine use in this population [89].

The ability to maintain adherence to daily oral medications while still in custody is already facilitated by several key factors: systems of control/coercion, stable housing, food security, access to medication regimens with minimal hurdles, and regular dispensing of medication directly to patients, obviating the need for transportation [90]. For these reasons, ART adherence and HIV viral suppression in the U.S. tend to improve when PWH are incarcerated compared with the periods preceding arrest and following reentry [52]. Hence, shifting patients from oral to LAI formulations may not improve viral suppression in custody. However, institutions with longer average stays may practically benefit from the implementation of LAI ART because medication administration in these settings is often carried out one or more times daily by medical staff (i.e., pill call). The lower personnel costs associated with bimonthly injections relative to daily pill administration may make LAIs more cost-effective than oral formulations, especially in carceral settings with high HIV prevalence.

Short-term incarcerations (< 30 days) pose a risk for HIV treatment disruption, one that may be avoided in patients who are on LAIs before entering a correctional facility [91]. Cabotegravir/rilpivirine injections may be difficult to initiate in jail or short-term treatment settings because of the several-week lead-in regimen. The lead-in will soon be optional, but the requirement for virologic suppression will remain; nevertheless, providers are known to use it off label for treatment-experienced patients who are not fully suppressed. However, if the oral bridge to the LAI formulation could be completed and the first (lead-in) injection administered shortly before release, LAIs for HIV might reduce the likelihood of post-release spikes of HIV viremia. A person’s ability to adhere to medication regimens is tested upon release, as the factors that help maintain it while incarcerated disappear and barriers to stability and routine emerge [91, 92]. For this reason, both individuals nearing reentry and their sex- and drug-using partners in the community may stand to gain the most benefit from LAI ART as they have the potential to maintain viral suppression and reduce forward HIV transmission during a critical period of potential increased risk of HIV transmission [93–95]. Analogously, the administration of opioid agonists (methadone and buprenorphine) at release has been shown in observational studies to reduce fatal overdose compared with no treatment [96].

## Interpersonal

### Providers

*Provider perceptions and readiness to prescribe LAIs differ by specialty*. Although initial studies of LAI antipsychotics showed a reduction in morbidity resulting from their use, they were not well received by psychiatrists who were concerned about increased side effects and patients’ ability to maintain therapeutic drug levels. Patients’ rights groups also argued that LAIs eliminate patient choice. However, beginning in the 1970s, studies increasingly showed that LAIs reduced relapse rates when compared with oral formulations, leading to the growing acceptance and use in the profession. Internationally, LAIs seem to be preferred to oral medication in some regions due to cultural perceptions that injected medications have greater potency [97].

The training required for providers to treat patients with MOUD, specifically buprenorphine, presents a barrier but does not currently differ between oral versus injectable formulations [98]. Despite guidelines that encourage LAI antipsychotic prescription based on patient preference at all stages of illness, provider misperceptions persist that LAIs are non-first-line agents to be reserved for patients with severe or resistant illness [99]. This misunderstanding creates an unnecessary barrier to access for patients who could benefit from early LAI use.

HIV providers may have the same perception and may not fully appreciate the benefits of prescribing LAI treatment. Finally, many rural settings lack HIV specialists, leading patients to receive care from non-specialists or to travel long distances for HIV care. Non-specialists may feel especially uncomfortable with providing LAIs for ART, and the need to travel long distances for treatment may make frequent clinic-based injections infeasible. Finally, the lack of Ryan White-funded providers in many rural areas means a lack of supportive services that could be marshaled to support consistent LAI use [100].

A significant lack of knowledge about and comfort with prescribing LAIs exists among prescribers. The most common reason patients are not given an LAI is that their provider fails to offer it [101]. As addiction medicine is a subspecialty, providers often lack the training required to address many aspects of OUD assessment and treatment and many patients with OUD never receive this level of care [102]. Provider stigma toward patients with OUD, including concerns about misuse and diversion of MOUD [103], can be as high or higher than among the general public [104]. Providers often overestimate the degree to which their patients are hesitant to try LAIs, which emphasizes the need for guidelines or requirements that providers routinely offer LAIs to patients. Indeed, an effect of direct-to-consumer pharmaceutical advertising is that patients become more proactive in requesting specific medications from providers who do not offer them upfront [105].

Education about the low likelihood of severe side effects, increased efficacy over oral medication, decreased re-hospitalization, and morbidity associated with LAIs over oral medications (especially in non-randomized controlled trials) are important and effective at increasing LAI use in mental health populations; employing these strategies with the rollout of HIV LAIs may help avoid some of these same pitfalls for PWH [81, 106–108].

### Patient Navigation

Wraparound services have long been available to PWH through the Ryan White HIV/AIDS Program [109], and peer navigation has been shown to be successful, particularly for populations vulnerable to incarceration [110]. Similar services are far less common for people experiencing mental illness and SUDs, and suggests that the rollout of LAIs for PWH may be more successful. This existing model of support for some PWH can be leveraged to include expanded patient navigation services to facilitate linkages, retention, and adherence to LAI ART. Successful approaches to complement patient navigation services include monetary incentives (both for HIV treatment [111] and for abstinence from drugs) for PWH [112] and mobile applications that provide support, motivation, and education regarding ongoing engagement in HIV treatment [113]. The current formulation for LAI ART requires much more frequent visits than the standard of care for patients on oral treatments, which is generally twice per year. The FDA has approved LAI ART for bimonthly injections, though some may continue to choose monthly injections. In either case, additional patient support is warranted.

## Patients

*Burdens on patient access and the need for patient education are similar between the three specialties*. In the clinician-authors experiences’, several factors associated with patient choice regarding LAIs for mental illness and SUD may apply to PWH as well. They include fear of needles, negative associations with injection drug use especially for those in recovery, concerns about control (such as the irreversibility of the injection), coercion, and loss of bodily integrity. The complexity of switching to an LAI regimen also may dissuade patients. Finally, the requirement that LAI ART is administered via gluteal injections may prevent use in transgender and cis-gender women who have or want these implants. As of this writing, anterior thigh injections are pending FDA approval.

Some people who use opioids report that long-acting buprenorphine would be more convenient and discreet than using daily opioid agonist therapy, particularly if the former is made available outside of the pharmacy or drug treatment setting that they associated with their OUD [84, 114, 115]. LAI antipsychotics have historically lasted 1 month, requiring more frequent patient visits than the standard 3-month visit interval for many psychiatric patients [12]. However, newer LAI antipsychotics have an increased duration of action to 3–6 months [12], lowering patient costs associated with more frequent visits and travel. Additionally, LAI antipsychotics have been associated with increases in adherence versus oral medication that range from 13 to 40% [116–118]. Rehospitalization, all-cause mortality, and symptoms have been shown to occur less frequently with LAI antipsychotics vs. oral medications [106, 108, 119, 120].

LAI antipsychotics uptake by patients has been supported by collaborative approaches [121] to discussing the option with patients, including addressing patients’ concerns and the stigma associated with LAIs [26, 101]. Integration of OUD treatment into primary care using a collaborative care intervention resulted in improved patient outcomes [122]. Providers also should address patients’ potential concerns about coercion, emphasize how LAIs provide better control over their illness than oral medication, and explain that their recommendation for LAI use is not based on disease severity [123]. The option of LAIs may improve medication uptake by offering more medication options to suit patient preferences.

Much to many providers’ surprise, many patients in clinical trials of cabotegravir/rilpivirine found injectable monthly ART preferable to daily oral ART and well-tolerated [124]. The most commonly reported side effects centered around pain or tenderness at the injection sites that were most commonly mild to moderate and short-lived. A smaller number of patients had more prolonged or more severe reactions at the site of the injection, and even fewer found the reactions sufficiently intolerable to stop taking the injections [125, 126]. Qualitative research into LAI PrEP and ART has consistently shown patient interest in LAI despite side effects, including among racial, sexual, and gender minority groups [127–130].

Education is particularly important for patients moving to LAIs from oral formulations as the two treatment approaches operate differently at the physiologic level. Low health literacy is distributed among regions and specific groups in the U.S. in a similar pattern to the HIV epidemic [131]. However, age and racial disparities in the continuum of care persist after accounting for health literacy, suggesting that insufficient education is one of several factors at work [132] (Table 1).

**Table 1:** Summary comparison across specialty references correspond to the reference number in the manuscript

Level	Issue	HIV context	MH/SU context	Implications for equitable LAI uptake for HIV treatment
Policy	Cost effectiveness	The cost of LAIs and several first-line oral HIV medications are comparable	Anti-psychotic LAI medications are associated with better health outcomes when compared to oral formulations [24]. High up-front cost is offset by lower subsequent costs in the form of hospitalizations rates and inpatient stays [25, 26]	More research is needed to establish cost effectiveness. This includes costs associated with onward horizontal and vertical transmission because of poor adherence [38, 39], hospitalizations and mortality. Additionally, costs are a shifting landscape as cost of oral formulations decline as patents expire
	Insurance Coverage	Cost containment measures will likely be an issue for HIV LAIs	Experience with LAI antipsychotics experience indicates that prior authorization, step therapy, and formulary exclusions, which can impede appropriate LAI prescribing [46]	Existing protections against cost containment for oral PrEP will need to include LAI formulations regardless of health coverage provider
	Medicaid Inclusion Act during Incarceration	The Medicaid Exclusion Act limits use of more expensive LAI formulations during confinement and reentry	Barriers related to the Medicaid Exclusion Act have been successfully overcome in MH/SU contexts	Efforts to eliminate the Medicaid Exclusion Act and exploration of options to suspend rather than terminate Medicaid during incarceration are opportunities in the HIV context
Community	Stigma and Marginalization of People living with Specific Health Conditions	Like mental illness and SUD, HIV is a highly stigmatized condition disproportionately affecting those experiencing marginalization. Clinicians and systems of care have contributed to the stigma [57]	Historically, LAIs for mental illness may have been preferentially used, likely influenced by intersectional stigma. This includes potential racial differences of use including increasing use by Black patients [63, 64]. In SU, stigma towards patients can be high or higher than among the general public [104]	An ongoing need for multilevel educational efforts exists, to normalize and destigmatize LAI use. Target media portrayals to influence patients directly and to ensure that policies do not reinforce stigma and inequity in administration
	Medical mistrust	Medical mistrust and conspiracy beliefs are rational responses to engaging hostile systems of care [70]. HIV-specific concerns include particularized fears related to needles and government actors’ intentional efforts to infect people with the virus	Potential racial differences in use of psych LAIs contribute to concerns and ethics [57] and targeted use among groups experiencing marginalization has created additional barriers	Recognize potential increased concerns about coercion and medical mistrust among affected subpopulations. Address medical mistrust head on
Organizational	Infrastructure	HIV system benefits from existing care delivery networks such as the Ryan White HIV/AIDS program. Capacity to deliver LAIs for HIV to be determined	In MH context, providers lack capacity to directly deliver injections because they lack nursing staff. In SU context, most settings have staff capacity to offer injections	Similar to the SU context, personnel scope of practice is unlikely to be a significant problem. However, the increase in required visit frequency must be addressed. Increasing access points (e.g. pharmacist delivery), addressing differences between private and public practice settings, and rural gaps in HIV clinical sites and personnel is needed
	Custody Settings	Use of LAI ART in custody to be determined. It may reduce the likelihood of post-release spikes of HIV viremia, a critical period of increased HIV transmission risk [93–95]	The use of psych LAIs are being explored by some locales and jail settings [83]. LAIs for SU are available in at least one prison within 90% of prison systems and 62% have at least one prison that offers three different SU medications [56]	Given successful implementation of LAIs for mental illness and SU in custodial settings, there is a need to plan for implementation of LAI administration to treat HIV in carceral settings where confinement is long enough to allow for med bridging. But *see* Patient-level issues
Interpersonal	Provider Perceptions	To be determined. Non-specialists may feel especially uncomfortable with providing LAIs for ART, and the need to travel long distances for treatment may make frequent clinic-based injections infeasible	Misunderstanding persists among MH providers. While acceptance has grown, some believe LAIs are non-first-line agents to be reserved for patients with severe or resistant illness [99]	HIV providers may not yet have negative/inaccurate perceptions but without targeted education, they may perceive LAIs for HIV in a similarly problematic manner as is true for MH context (e.g. provider failure to offer as the reason why patients are given LAI)
Patients	Patient Perceptions	Many patients in clinical trials of cabotegravir/rilpivirine found injectable monthly ART preferable and well tolerated when compared to daily oral ART [124]	Use has been supported by collaborative approaches where providers address patients’ concerns directly and focus on reducing LAI stigma [26, 108, 119]. LAIs for SU may trigger fear of needles for those with a history of injection drug use. However, some people who use opioids report that long-acting buprenorphine would be more convenient or discreet than using daily opioid agonist therapy [84, 114, 115]	Future efforts should learn from the collaborative approaches used in the MH context. Ongoing need for multilevel educational efforts must reach patients and their loved ones. Targets should look to addressing distrust and LAI stigma as well as physiologic differences between the two treatment approaches
	Patient Access	Individuals facing adverse social and structural barriers (e.g., housing instability [17, 18], poverty [19], criminal justice involvement [20], intimate partner violence [22], along with SUDs [23]) are at increased risk for suboptimal retention in care and poor HIV medication adherence and persistence	LAIs for mental illness have historically required more frequent patient visits than oral formulations [12]. Newer formulations last 3–6 months, however, lowering patients’ cost associated with more frequent visits, including travel [12]	Given the adverse social and structural barriers many PWH face, increased frequency of clinic visits must addressed. For those facing health conditions at the intersection of HIV, there remain opportunities to develop innovative approaches including co-located administration of injectable medications to address multiple health needs

*ART* antiretroviral therapy; *LAI* long acting injectable; *MH* mental health; *PrEP* pre-exposure prophylaxis (for prevention of HIV); *SU* substance use; *SUD* substance use disorder

## Future Considerations

The rollout of a new treatment modality often faces challenges in finding acceptance among medical practitioners and the public. In the case of LAIs for antipsychotics, providers feared that the medications would fail to achieve a therapeutic dose and cause unacceptable side effects. Advocacy groups in the community used terms like “chemical straitjacket”, asserting LAIs would be used to impose treatment upon patients regardless of their preferences [133].

Even once a level of acceptance is achieved, barriers highlighted in this paper must be addressed early to both minimize negative perceptions and ensure access to those patients who have the greatest potential to benefit from long-acting formulations. The populations most affected by the HIV epidemic are also the populations most likely to both lack access to and mistrust the treatment in the first place [134]. Community engagement and effective messaging are critical [71], but are undermined without policies to ensure provider capacity and enough insurance coverage to get the medications to the patients who are asked to adhere to the regimen and told to trust that the system will work for them. This is a critically important objective at a time when medical mistrust is increasing [134]. Otherwise, many of the above challenges also have the potential to negatively impact the rollout of LAI ART and to widen disparities in HIV outcomes.

Cost-effectiveness analyses have proven useful in encouraging policy and health system support for LAI use for the treatment of mental illness. Additional cost-effectiveness analyses of LAI ART could inform recommendations regarding priorities for expanding access to LAIs for ART. However, even if LAIs prove to be cost-effective for the treatment of HIV infection, the potentially higher upfront cost may impede their dissemination. As demonstrated in other contexts, when LAIs are covered as a *medical* benefit, insurers may implement cost-containment strategies, such as requiring prior authorization or step therapy [46]. Insurance coverage raises several important questions to address moving forward: Will patient cost sharing require a coinsurance based on the expensive list prices for LAIs or will lower cost copayment be allowed? What will be the cost associated with monitoring LAI use over time? Will pharmaceutical company rebates apply to LAIs, as they do for oral medications? The December 2021 FDA approval of cabotegravir for HIV pre-exposure prophylaxis [135]; heightens the urgency of ensuring an equitable and efficient rollout of LAI ART.

## Data Availability

N/A.

## Code Availability

N/A.
